# Incidence of *Clostridioides difficile* infection (CDI) related to antibiotic prescribing by GP surgeries in Wales

**DOI:** 10.1093/jac/dkab204

**Published:** 2021-06-21

**Authors:** Florence Tydeman, Noel Craine, Kimberley Kavanagh, Helen Adams, Rosy Reynolds, Victoria McClure, Harriet Hughes, Matt Hickman, Chris Robertson

**Affiliations:** 1 Department of Mathematics and Statistics, University of Strathclyde, Glasgow G1 1XH, Scotland; 2 CDSC, Public Health Wales, Ysbyty Gwynedd, Bangor LL57 2PW, Wales; 3 Betsi Cadwaladr University Health Board, Ysbyty Gwynedd, Bangor LL57 2PW, Wales; 4 Population Health Sciences, Bristol Medical School, University of Bristol BS8 2PS, England; 5 Public Health Wales, Microbiology, University Hospital of Wales, Cardiff CF14 4XW, Wales; 6 Health Protection Scotland, Glasgow G2 6QE, Scotland

## Abstract

**Background:**

*Clostridioides difficile* infection (CDI) is a healthcare-acquired infection (HAI) causing significant morbidity and mortality. Welsh CDI rates are high in comparison with those in England and Scotland.

**Objectives:**

This retrospective ecological study used aggregated disease surveillance data to understand the impact of total and high-risk Welsh GP antibiotic prescribing on total and stratified inpatient/non-inpatient CDI incidence.

**Methods:**

All cases of confirmed CDI, during the financial years 2014–15 to 2017–18, were linked to aggregated rates of antibiotic prescribing in their GP surgery and classified as ‘inpatient’, ‘non-inpatient’ or ‘unknown’ by Public Health Wales. Multivariable negative-binomial regression models, comparing CDI incidence with antibiotic prescribing rates, were adjusted for potential confounders: location; age; social deprivation; comorbidities (estimated from prevalence of key health indicators) and proton pump inhibitor (PPI) prescription rates.

**Results:**

There were 4613 confirmed CDI cases, with an incidence (95% CI) of 1.44 (1.40–1.48) per 1000 registered patients. Unadjusted analysis showed that an increased risk of total CDI incidence was associated with higher total antibiotic prescribing [relative risk (RR) (95% CI) = 1.338 (1.170–1.529) per 1000 items per 1000 specific therapeutic group age-sex related GP prescribing units (STAR-PU)] and that high-risk antibiotic classes were positively associated with total CDI incidence. Location, age ≥65 years and diabetes were associated with increased risk of CDI. After adjusting for confounders, prescribing of clindamycin showed a positive association with total CDI incidence [RR (95% CI) = 1.079 (1.001–1.162) log items per 1000 registered patients].

**Conclusions:**

An increased risk of CDI is demonstrated at a primary care practice population level, reflecting their antibiotic prescribing rates, particularly clindamycin, and population demographics.

## Introduction

*Clostridioides difficile* is a bacterium that colonizes the bowel in approximately 5% of adults.^1^ In those who develop symptomatic *C. difficile* infection (CDI), diarrhoea, fever and abdominal pain are common. The majority of reported CDI cases are related to a hospital or care-home stay; however, recent studies indicate that the incidence of community-associated *C. difficile* infection (CA-CDI) is increasing and may account for up to 30% of all CDI cases.^2^^,^^3^ CA-CDI population-based studies report similar risk factors to hospital-associated (HA-CDI) studies; however, there have been key differences reported, with CA-CDI cases linked to younger patients and less severe illness.^3^

Surveillance data in Wales indicate that CDI rates are high in comparison with England and Scotland: 36.7 CDI per 100 000 population in Wales compared with 23.9 CDI per 100 000 in England and 30.1 CDI per 100 000 in Scotland (2017).^4–6^ CDI in hospitalized patients results in poorer patient outcomes, increased length of hospital stay and treatment costs.^7^^,^^8^ CDI has a significant effect on patient morbidity and mortality, associated with 30 day all-cause mortality rates of 9%–38% and attributable mortality rates of 5.7%–6.9%.^9^

Risk factors for CDI include antimicrobial exposure, age, proton pump inhibitor (PPI) exposure, previous stay in hospital/nursing home and comorbidities such as hypertension.^2^^,^^10^^,^^11^ The epidemiology of *C. difficile* may be shaped by a range of environmental factors such as high hospital bed occupancy rates;^12^ however, evidence from a broad range of studies supports the central role of antibiotic use as the key driver of symptomatic CDI.^13^

Associations between broad-spectrum antibiotic use and CDI have been consistently reported.^14–16^ Broad-spectrum antibiotics are likely to disrupt the microbial ecology of the gut, leading to an overgrowth of pre-existing previously asymptomatic *C. difficile* or a newly acquired organism. Antibiotic stewardship is now a core component of national responses to CDI. Current Welsh government policy encourages prudent and appropriate use of antibiotics.^17^ In primary care, this may be interpreted as encouraging low rates of antibiotic prescribing by raising the threshold for initiation of antibiotics or by delaying prescription. Recent research provides strong evidence that the declines in CDI observed in England are associated with changes in antibiotic use, particularly fluoroquinolone usage.^13^

Primary care antibiotic prescribing rates are falling; from 2013–14 to 2017–18, Welsh primary care prescribing saw an 11.9% reduction in the total number of items dispensed.^18^ Stewardship of particular broad-spectrum antimicrobials associated with a high risk of CDI is recommended to reduce the number of patients predisposed to CDI and lower transmission rates.^19^ The four broad-spectrum antibiotics targeted by stewardship programmes, collectively called the ‘4C antimicrobials’, are cephalosporins, clindamycin, ciprofloxacin and co-amoxiclav.^20^

The epidemiology of *C. difficile* is complex; observed patterns of disease may be due to the individual or combined effects of (i) outbreaks in healthcare settings; (ii) exposure to environmental sources of *C. difficile*; and (iii) triggering of recently acquired or long-term colonization by exposure to factors such as antibiotics that disrupt the gut microbiota.

The primary objective of this study was to understand the impact of total and high-risk Welsh GP antibiotic prescribing on total CDI incidence, with a secondary aim of stratifying CDI incidence by inpatient and non-inpatient cases.

## Methods

This was a retrospective ecological study of the incidence of CDI across Wales between the financial years 2014–15 and 2017–18 including all cases of laboratory-confirmed CDI, from routine surveillance data collated by Public Health Wales every financial year,^21^ linked to aggregated rates of antibiotic prescribing in the GP surgery at which the patient was registered.

### Data sources and linkage

All glutamate dehydrogenase (GDH)-positive/toxin-positive CDI cases reported to the national surveillance system for *C. difficile* infection were provided by Public Health Wales including, when available, the GP surgery at which the case was registered. Following linkage of patients to practices, and subsequent linkage of relevant practice-level data, the data were anonymized prior to analysis, including anonymizing the practice and health board in which the practice was based. Classification of patients into ‘inpatient’, ‘non-inpatient’ or ‘unknown’ was part of routine Public Health Surveillance activity using the following definitions: an ‘inpatient’ was associated with a sample submitted from a hospital inpatient location irrespective of specimen timing in relation to admission date. A ‘non-inpatient’ originated from a non-inpatient setting (GP, hospital, A&E or admission units, with no assessment being made of time elapsed since admission). For patients with more than one positive sample in a financial year, the first positive result for any one patient was selected. CDI cases were excluded if the patients were registered with a practice outside Wales or the practice was unknown.

The number of CDI cases for patients registered at each of the Welsh GP surgeries was aggregated for each financial year (2014–15, 2015–16, 2016–17, 2017–18). The GP location for each patient was obtained from the Welsh demographic system using the patient’s NHS number and GP surgery, allocated by Public Health Wales and the NHS Wales Informatics Service (NWIS).

Rates of antibiotic prescribing by practice were obtained from the Welsh pharmacy database Comparative Analysis System for GP Prescribing Audit (CASPA). Data were collated for all antibiotics and separately for those classes considered a high risk for CDI (cephalosporins, quinolones, co-amoxiclav and clindamycin). Total antibiotics were collated as items per 1000 specific therapeutic group age/sex-related GP prescribing units (STAR-PU); separate classes were collated as items per 1000 patients registered at the practice. The STAR-PU is a measure weighted to reflect the age and gender mix of the practice and specific drug type.^18^ Antibiotic GP prescribing was collated by financial year. Rates of PPI prescription were obtained as defined items per 1000 registered patients.

Attributes relating to GP surgeries and rates of CDI were collated independently for each of the four financial years. Practice population size data were obtained from the CASPA GP prescribing database for each financial year. Mergers and changes in practice structure over the 4 years explained the practice population variation during the analysis period. Data regarding the percentage of patients aged ≥65 years, in each practice, was obtained from the Public Health Wales Observatory. The social deprivation characteristic of each practice was estimated by the percentage of the practice population in the most deprived 40% of Wales lower super output area using the 2014 version of the Welsh indices of multiple deprivation and was used for subsequent study years.

The level of comorbidities of the practice populations was estimated from disease and risk behaviour-specific prevalence rates reported as part of the general medical services contract Quality and Outcome Framework (QOF). Data were extracted separately for each of the four periods due to variation in GP surgery profile between time periods. The following practice-level prevalence indicators were included in initial analyses: patients with COPD; patients ever diagnosed with established hypertension; and patients at least 17 years old diagnosed with a specified diabetes and by type. QOF data were generally available for 2014–15 and 2015–2016 but less so for 2016–17 and no data available for 2017–18 due to relaxed NHS Wales data capture requirements.^22^ Where data were missing for later years, previous practice measures were imputed using a last-one-carried-forward (LOCF) method.^23^ This assumes that practice population indicators remain similar between years.

### Ethics and data storage

The study did not require ethical approval as it used data on CDI cases per GP surgery, delinked from any identifiable individual patient, and publicly available GP practice-level data. In addition, GP surgery identity and health board were anonymized within the analysis dataset. Data are stored on a secure server of the University of Bristol, with access limited to a small number of study team members. This process was cleared by the Public Health Wales Information Governance team. Public Health Wales collates *C. difficile* data on an all-Wales basis for ongoing surveillance purposes.

### Statistical analysis

Negative-binomial regression models investigated the association between practice-level antibiotic GP prescribing and the outcome of CDI incidence in Welsh GP surgeries. Primary analysis assessed the association between total CDI incidence (inpatient/non-inpatient cases combined) and total and high-risk practice-level antibiotic prescribing. The associations with potential confounding variables (financial year, health board, age, social deprivation, PPI use, COPD, diabetes and hypertension) were examined and adjusted for in regression models. Boxplots were used to visualize differences in both inpatient and non-inpatient CDI incidences by quartiles of GP prescribing level.

For the primary analysis, a model including interaction terms between CDI case source (inpatient/non-inpatient) and all other covariates was examined. Backward selection was performed to identify any statistically significant terms. Interaction tests were used to examine the differences amongst CDI case source (inpatient or non-inpatient) and some covariates. The results of these interaction tests motivated a secondary analysis of CDI incidence, with stratification of inpatient and non-inpatient cases, modelled against total and high-risk antibiotic GP prescribing.

Natural log transformations were applied to prescribing rates for co-amoxiclav, cephalosporins, clindamycin and quinolones, as the distribution of rates were heavily skewed, therefore the units are expressed as log items per 1000 registered patients. Incidence rates are presented with 95% CIs, calculated using Byar’s approximation.^24^ Statistical analysis was performed using R 3.5.1.

## Results

### CDI incidence and antibiotic prescribing summary

There were 4613 total confirmed CDI cases from 2014–15 to 2017–18 linked to GP surgeries over seven Welsh health boards, serving a population of over 3 million: incidence (95% CI) was 1.44 (1.40–1.48) cases per 1000 registered patients. The number of inpatient and non-inpatient cases were 2580 (61.8%) and 1763 (38.2%), with incidences (95% CI) per 1000 patients of 0.89 (0.86–0.92) and 0.55 (0.53–0.58), respectively. There was a 15.6% decrease in total CDI incidence from 2014–15 to 2017–18, from 0.40 (0.38–0.43) to 0.34 (0.32–0.36) cases per 1000 registered patients; however, 2017–18 showed a 7.6% increase in CDI incidence relative to 2016–17. The inpatient and non-inpatient split of CDI cases remained largely unchanged throughout the study period (Table 1).

**Table 1.** CDI incidence rates by financial year

	Financial year			
	2014–15	2015–16	2016–17	2017–18
CDI cases (95% CI) per 1000 registered patients				
Total	0.403 (0.381–0.425)	0.381 (0.360–0.403)	0.316 (0.297–0.336)	0.340 (0.320–0.361)
Inpatient	0.257 (0.240–0.275)	0.232 (0.215–0.249)	0.195 (0.180–0.211)	0.206 (0.191–0.222)
Non-inpatient	0.146 (0.133–0.160)	0.149 (0.136–0.163)	0.121 (0.110–0.134)	0.134 (0.122–0.147)
GP surgeries, *n*	460	451	439	426

Median total antibiotic prescribing rates fell by 11.3% from 2014–15 to 2017–18 and consistently decreased each year. Prescribing rates for co-amoxiclav, cephalosporins and quinolones also decreased during this time with co-amoxiclav and cephalosporins displaying similar median prescribing rates throughout (29.22 and 27.15 items per 1000 in 2014–15 then 20.65 and 18.45 items per 1000 in 2017–18), whereas the rate of quinolone prescribing was lower (14.49 items per 1000 in 2014–15 then 12.39 items per 1000 in 2017–18). In comparison with all other high-risk antibiotic groups, clindamycin was rarely prescribed; however, it was the only antibiotic group seen to increase each year (0.75 items per 1000 in 2014–15 then 1.04 items per 1000 in 2017–18) (Table 2). The overall total GP antibiotic prescribing rates varied between surgeries and years; the median (IQR and min–max) prescribing rate was 1272 (1077–1450 and 374–2677) items per 1000 STAR-PU from 2014–15 to 2017–18. Overall high-risk antibiotic prescribing also varied between surgeries and years, with minimum GP prescribing rates of <1 item per 1000 for all four high-risk antibiotics. Co-amoxiclav, cephalosporin, clindamycin and quinolone median (IQR and min–max) prescribing rates were 23.3 (15.6–33.7 and 0.6–226.8), 22.2 (13.4–33.8 and 0.00–143.7), 0.9 (0.4–2.0 and 0.00–14.1) and 13.3 (9.6–17.5 and 1.0–67.6) items per 1000 registered patients, respectively.

**Table 2.** Antibiotic prescribing rates for total antibiotics, co-amoxiclav, cephalosporins, clindamycin and quinolones by financial year

Antibiotics	Financial year			
	2014–15	2015–16	2016–17	2017–18
Total, median (IQR) items per 1000 STAR-PU	1353 (1157–1557)	1278.6 (1081.6–1453.6)	1230.4 (1049.3–1392.1)	1199.8 (1043.7–1363.0)
Co-amoxiclav, median (IQR) items per 1000 registered patients	29.22 (19.20–41.26)	23.81 (15.76–33.97)	21.59 (14.73–30.14)	20.65 (14.04–27.95)
Cephalosporins, median (IQR) items per 1000 registered patients	27.15 (18.27–40.27)	23.65 (14.74–34.59)	20.81 (12.12–29.70)	18.45 (11.04–27.86)
Clindamycin, median (IQR) items per 1000 registered patients	0.75 (0.27–1.81)	0.88 (0.31–1.84)	0.97 (0.44–2.08)	1.04 (0.39–2.02)
Quinolones, median (IQR) items per 1000 registered patients	14.49 (10.77–19.12)	13.13 (9.62–17.33)	12.97 (9.59–16.73)	12.39 (8.82–16.77)

### Total CDI incidence

Unadjusted models for the primary analysis showed increased risk of total CDI incidence with increasing percentage of practice population comorbidities [for a 1% increase in the percentage of the practice population with COPD, relative risk (RR) (95% CI) = 1.173 (1.120–1.230); with diabetes, RR (95% CI) = 1.152 (1.114–1.191); and with hypertension, RR (95% CI) = 1.055 (1.043–1.068)]. An increased risk in total CDI was also associated with increasing percentage of patients aged ≥65 years [RR (95% CI) = 1.037 (1.029–1.045)] and PPI prescribing (per 1000 items per 1000 patients) [RR (95% CI) = 1.018 (1.011–1.025)] (Table 3).

**Table 3.** Unadjusted and adjusted RRs of total CDI incidence compared with total antibiotic prescribing

	Unadjusted	Adjusted	
	RR (95% CI)	RR (95% CI)	*P* value
Total antibiotics (per 1000 items per 1000 STAR-PU)	1.3375 (1.1696–1.5293)	1.1413 (0.9714–1.3404)	0.108
Financial year			
2014–15	1	1	—
2015–16	0.9453 (0.8518–1.0490)	0.9397 (0.8518–1.0365)	0.214
2016–17	0.7901 (0.7095–0.8798)	0.7823 (0.7056–0.8672)	<0.001
2017–18	0.8388 (0.7541–0.9330)	0.8257 (0.7455–0.9144)	<0.001
Health boards (1–7)	—	—	<0.001
Patients aged ≥65 years (per 1% increase)	1.0370 (1.0293–1.0447)	1.0269 (1.0134–1.0407)	0.001
Social deprivation score (per 1% increase)	0.9995 (0.9980–1.0009)	1.0002 (0.9980–1.0025)	0.856
PPI (per 1000 items per 1000 patients)	1.0175 (1.0105–1.0246)	0.9967 (0.9877–1.3404)	0.481
COPD (per 1% increase)	1.1732 (1.1196–1.2292)	1.0594 (0.9906–1.1322)	0.093
Diabetes (per 1% increase)	1.1518 (1.1138–1.1913)	1.0609 (1.0023–1.1227)	0.039
Hypertension (per 1% increase)	1.0550 (1.0426–1.0676)	1.0051 (0.9841–1.0264)	0.639

Unadjusted models include only one predictor variable (univariate analysis). Adjusted models are adjusted for financial year, health board, percentage of patients aged ≥65 years, social deprivation score (percentage of registered patients living in the most deprived areas), PPI (per 1000 items per 1000 registered patients) and percentage of patients with COPD, diabetes and hypertension.

Total CDI incidence was associated with higher total antibiotic prescribing [RR (95% CI) = 1.338 (1.170–1.529) per 1000 items per 1000 STAR-PU] (Table 3). High-risk antibiotic classes were also seen to be positively associated with total CDI incidence; co-amoxiclav, clindamycin, cephalosporins and quinolones presented a 12.0%, 14.9%, 24.6% and 28.0% increase in risk of CDI per unit increase in log items per 1000 registered patients (Table 4).

**Table 4.** Unadjusted and adjusted RR of total CDI incidence compared with rates of pre-defined high-risk antibiotic groups

	Unadjusted	Adjusted	
High-risk antibiotic GP prescribing (log items per 1000)	RR (95% CI)	RR (95% CI)	*P* value
Co-amoxiclav	1.1200 (1.0451–1.2005)	1.0803 (0.9993–1.1682)	0.054
Cephalosporins	1.2456 (1.1715–1.3250)	1.0638 (0.9858–1.1485)	0.111
Clindamycin	1.1485 (1.0707–1.2319)	1.0787 (1.0014–1.1618)	0.046
Quinolones	1.2798 (1.1690–1.4015)	1.0125 (0.9180–1.1168)	0.805

Adjusted models are adjusted for financial year, health board, percentage of patients aged ≥65 years, social deprivation score (percentage of registered patients living in the most deprived areas), PPI (per 1000 items per 1000 registered patients) and percentage of patients with COPD, diabetes and hypertension.

The fully adjusted model for the primary analysis showed diminished effects and wider 95% CIs for total CDI incidence with total antibiotic prescribing [RR (95% CI) = 1.141 (0.971–1.340) per 1000 items per 1000 STAR-PU]. Higher percentage of practice population aged ≥65 years and with diabetes were both associated with increased total CDI incidence, and varied between health boards and financial years (Table 3). Higher prescribing of clindamycin was associated with high total CDI incidence [RR (95% CI) = 1.079 (1.001–1.162) per log items per 1000 registered patients] in the fully adjusted model. The other high-risk antibiotics (co-amoxiclav, cephalosporins and quinolones) showed positive associations with total CDI incidence; however, results were weaker with wider 95% CIs [RR (95% CI) = 1.080 (0.999–1.168), 1.063 (0.986–1.149) and 1.013 (0.918–1.117), respectively] (Table 4).

**Figure 1.**
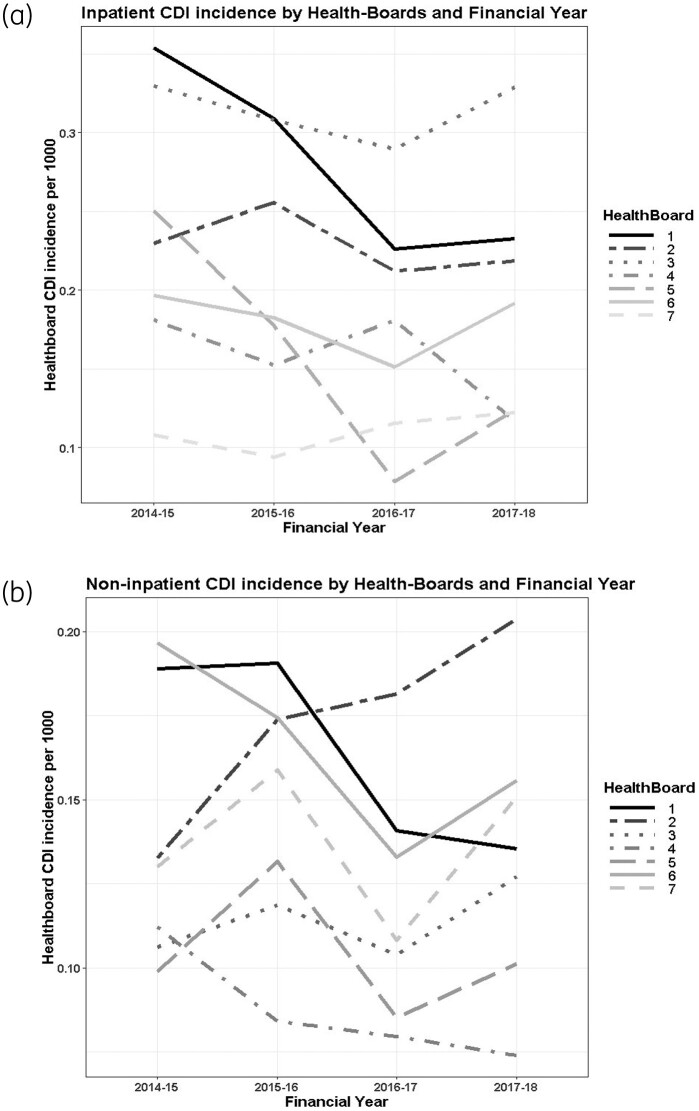
Health board CDI incidence per 1000 registered patients by financial year (2014–15 to 2017–18). (a) Inpatients; (b) non-inpatients.

An interaction model for total CDI incidence showed the relationship with inpatient or non-inpatient cases to vary for deprivation and health board (tests for interaction *P* = 0.034 and *P* < 0.001, respectively). Figure 1(a and b) shows that inpatient and non-inpatient CDI incidence varied between health boards and over time; however, the health boards were not consistent with one another.

### Stratified CDI incidence

The secondary analysis showed inpatient CDI to be weakly associated with total antibiotic prescribing (per 1000 items per 1000 STAR-PU) [RR (95% CI) = 1.101 (0.907–1.335)], with non-inpatient CDI incidence showing similar, but slightly higher, results [RR (95% CI) = 1.213 (0.942–1.560)]. Inpatient CDI incidence showed an association with financial years. Each year was lower in comparison with 2014–15; however, this was not seen for non-inpatient cases. Inpatient CDI incidence was also seen to be associated with the percentage of the practice population with diabetes and aged ≥65 years [RR (95% CI) = 1.079 (1.008–1.155) and 1.034 (1.018–1.050), respectively] (Table 5).

**Table 5.** Adjusted RRs of inpatient and non-inpatient CDI incidence compared with total antibiotic prescribing

	Inpatients		Non-inpatients	
	RR (95% CI)	*P* value	RR (95% CI)	*P* value
Total antibiotics (per 1000 items per 1000 STAR-PU)	1.1010 (0.9070–1.3353)	0.329	1.2128 (0.9418–1.5596)	0.13
Financial year				
2014–15	1	—	1	—
2015–16	0.8884 (0.7902–0.9987)	0.0479	1.0340 (0.8872–1.2051)	0.668
2016–17	0.7482 (0.6613–0.8462)	<0.001	0.8430 (0.7171–0.9906)	0.037
2017–18	0.7785 (0.6889–0.8795)	<0.001	0.9173 (0.7824–1.0752)	0.287
Health boards (1–7)	—		—	<0.001
Patients aged ≥65 years (per 1% increase)	1.0339 (1.0176–1.0504)	—	1.0152 (0.9941–1.0367)	0.161
Social deprivation score (per 1% increase)	1.0014 (0.9987–1.0041)	0.297	0.9985 (0.9950–1.0020)	0.39
PPI (per 1000 items per 1000 patients)	0.9981 (0.9871–1.0092)	0.734	0.9946 (0.9804 –1.0090)	0.453
COPD (per 1% increase)	1.0532 (0.9712–1.1411)	0.21	1.0608 (0.9536–1.1782)	0.274
Diabetes (per 1% increase)	1.0794 (1.0082–1.1552)	0.027	1.0317 (0.9437–1.1272)	0.488
Hypertension (per 1% increase)	0.9949 (0.9700–1.0203)	0.329	1.0240 (0.9906–1.0582)	0.158

Adjusted models are adjusted for financial year, health board, percentage of patients aged ≥65 years, social deprivation score (percentage of registered patients living in the most deprived areas), PPI (per 1000 items per 1000 registered patients) and percentage of patients with COPD, diabetes and hypertension.

There was a positive association with all high-risk antibiotic groups for both inpatient and non-inpatient CDI cases, apart from non-inpatient cases with quinolone prescribing. Estimates were consistent for inpatients/non-inpatients and comparable with total CDI estimates, although with wider 95% CIs (Table 6).

**Table 6.** Adjusted RRs of inpatient and non-inpatient CDI incidence associated with rates of pre-defined high-risk antibiotic groups

	Inpatient CDI incidence		Non-inpatient CDI incidence	
log items per 1000	RR (95% CI)	*P* value	RR (95% CI)	*P* value
Co-amoxiclav	1.0768 (0.9804–1.1834)	0.124	1.0785 (0.9546–1.2197)	0.230
Cephalosporins	1.0634 (0.9700–1.1665)	0.189	1.0655 (0.9466–1.2007)	0.298
Clindamycin	1.0827 (0.9902–1.1837)	0.081	1.0716 (0.9540–1.2035)	0.242
Quinolones	1.0268 (0.9121–1.1562)	0.663	0.9873 (0.8478–1.1504)	0.869

Adjusted models are adjusted for financial year, health board, percentage of patients aged ≥65 years, social deprivation score (percentage of registered patients living in the most deprived areas), PPI (per 1000 items per 1000 registered patients) and percentage of patients with COPD, diabetes and hypertension.

**Figure 2.**
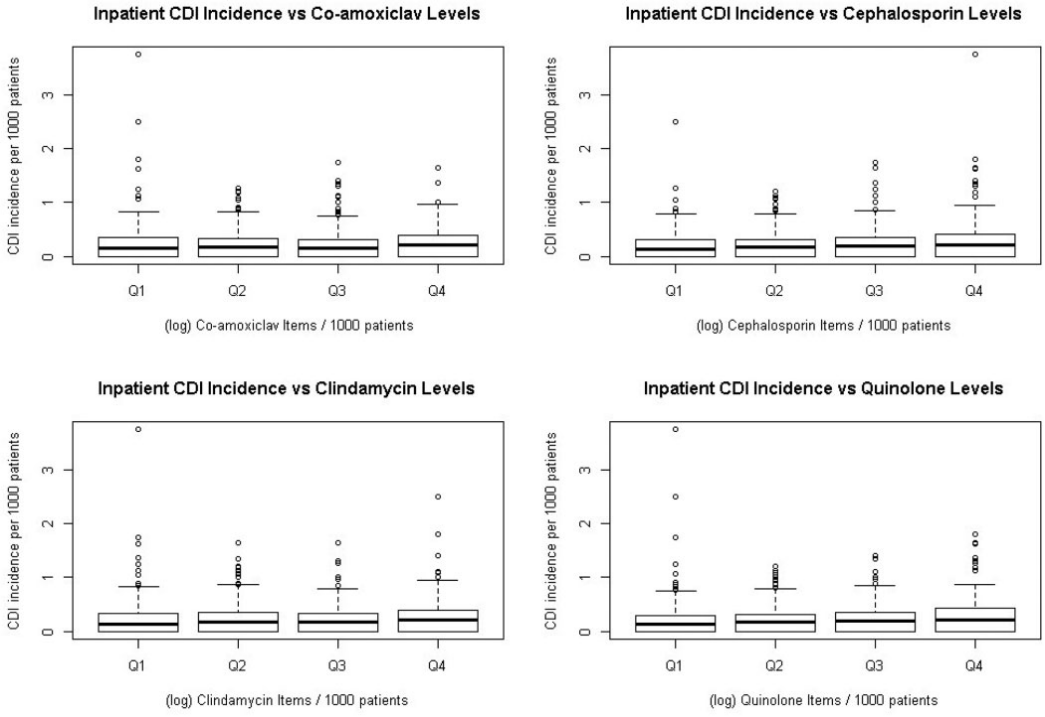
Inpatient CDI incidence per 1000 registered patients by quartiles (Q) of each high-risk antibiotic group prescribing rates (Q1–Q4) at Welsh GP surgeries (log items per 1000 registered patients). Q1 represents the GP surgeries with the lowest 25% prescribing rates for each high-risk antibiotic and Q4 represents those with the highest 25% prescribing rates.

**Figure 3.**
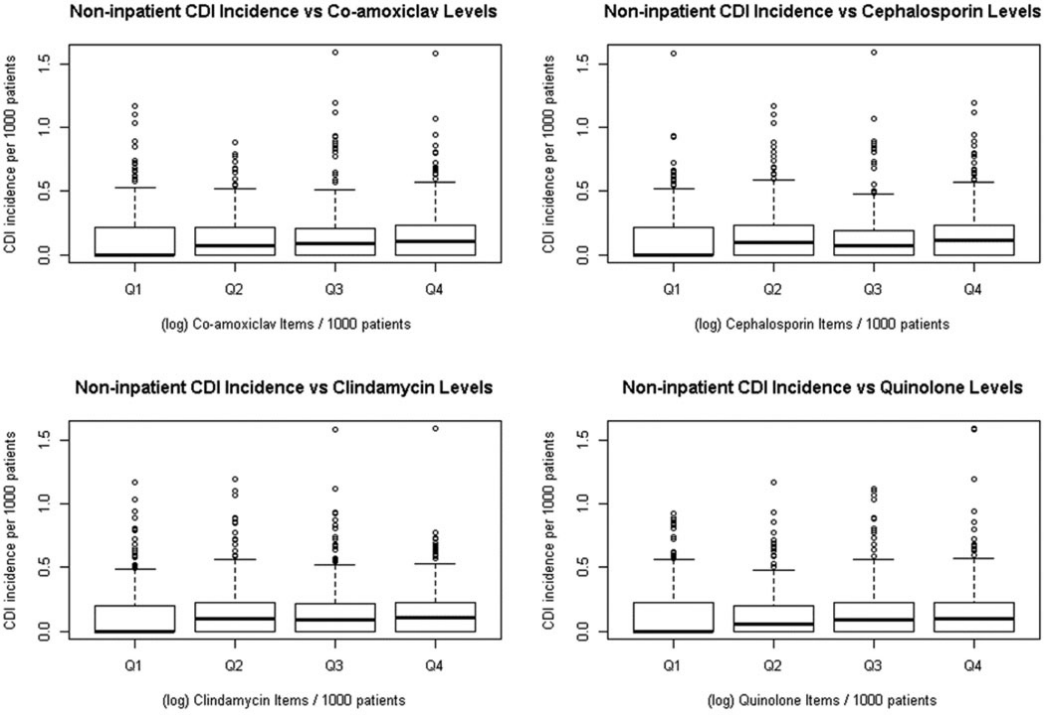
Non-inpatient CDI incidence per 1000 registered patients by quartiles (Q) of each high-risk antibiotic group prescribing rates (Q1–Q4) at Welsh GP surgeries (log items per 1000 registered patients). Q1 represents the GP surgeries with the lowest 25% prescribing rates for each high-risk antibiotic and Q4 represents those with the highest 25% prescribing rates.

A gradual increase in median inpatient CDI incidence was observed in the boxplot in Figure 2, across increasing quartiles of high-risk antibiotic (co-amoxiclav, cephalosporin, clindamycin and quinolone) prescribing (quartile 1–quartile 4), with quartile 4 showing the widest variability. For non-inpatient CDI there was an increase in median incidence from quartile 1 prescribing compared with all other prescribing categories (quartile 2, quartile 3 and quartile 4) for all high-risk antibiotic classes (Figure 3).

## Discussion

### Key findings

This retrospective ecological study in Wales confirmed the hypothesis that overall GP surgery-level antibiotic prescribing rates were associated with an elevated risk of CDI. Unadjusted analysis showed a higher risk of total CDI incidence with total practice antibiotic prescribing [RR (95% CI) = 1.337 (1.170–1.529) per 1000 items per 1000 STAR-PU]; however, this effect was lower after adjusting for practice demographic covariates [RR (95% CI) = 1.141 (0.971–1.340)]. This approximates to a 10% increase in risk of CDI between first- and third-quartile prescribers in 2017–18. Even after accounting for practice demographics, there could still be a 5% increase in risk. Higher total CDI incidence was associated with high percentages of practice population aged ≥65 years and with diabetes. Incidence also varied between health boards and decreased between 2014–15 and 2017–18. An increased risk of total CDI incidence was associated with antibiotic groups known to be high risk for CDI (co-amoxiclav, clindamycin, quinolones and cephalosporins) in the unadjusted analysis. An elevated risk of CDI was associated with clindamycin after adjusting for covariates [RR (95% CI) = 1.079 (1.001–1.162) per log items per 1000 registered patients], relating to a 4% increased risk between first- and third-quartile prescribers in 2017/18. Effects weakened for all other high-risk groups after adjusting for confounders. The secondary analyses indicated an increased risk of both inpatient and non-inpatient CDI associated with higher total antibiotic prescribing. High-risk antibiotic groups also suggested increased risk for both inpatient and non-inpatient CDI. Evidence was weaker in the secondary analyses as statistical power was lost by stratification.

### Comparative analysis

There was an overall incidence rate of 1.44 (95% CI 1.40–1.48) per 1000 patients for total CDI, across four financial years. Yearly total CDI incidence decreased by 15.6% from 2014–15 to 2017–18 but increased between 2016–17 and 2017–18. Overall, antibiotic prescribing rates fell by 11.3% from 2014–15 to 2017–18, comparable with Public Health Wales reports, presumably in response to antibiotic stewardship efforts.^18^ Prescribing rates of high-risk antibiotics also decreased during this time, by 29.3%, 32.0% and 14.5% for co-amoxiclav, cephalosporins and quinolones, respectively. Clindamycin prescribing rates were seen to be low (≤1.04 item per 1000 registered patients) for each financial year, reflecting restricted indications in primary care guidelines; however, they increased by 38% from 2014–15 to 2017–18 (0.75 items per 1000 to 1.04 items per 1000). The reasons for this isolated increase are not clear. Common reasons for prescribing clindamycin include skin and soft tissue infection (including MRSA) and diabetic foot infection, particularly in the context of penicillin allergy, for which alternative appropriate antibiotics could be considered.^19^^,^^25^ Penicillin allergies may provide the reasoning behind high-risk antimicrobial prescribing such as clindamycin; however, inaccurate records of penicillin allergies can lead to unnecessary prescribing of such antibiotics.^26^ Patients with a noted penicillin allergy are more likely to be prescribed clindamycin and experience worse health outcomes.^27^ Penicillin allergies show an increased risk of MRSA and *C. difficile* [HR (95% CI) = 1.69 (1.51–1.90) and 1.26 (1.12–1.40), respectively] alongside increased use of macrolides, clindamycin and fluoroquinolones.^28^ Improving the accuracy of recording penicillin allergy labels may be a good target for improving antibiotic stewardship and, in turn, affecting incidence of *C. difficile*.

The risks of CDI associated with the 4C antibiotic group are widely recognized and considered in antibiotic stewardship frameworks.^29^ A meta-analysis of the association between CA-CDI and antibiotics identified clindamycin to have the strongest association with CA-CDI [OR (95% CI) = 20.43 (8.50–49.09), followed by fluoroquinolones and cephalosporins [5.65 (4.38–7.28) and 4.47 (1.60–12.50), respectively].^30^ The impact of the lasting effects of 4C prescribing can be seen in the risk of CA-CDI; a population-based case–control study on the cumulative and temporal effects of antimicrobial prescribing on CA-CDI showed that individuals exposed to ≥29 DDDs of any high-risk antimicrobial (cephalosporins, clindamycin, co-amoxiclav and quinolones) had an OR (95% CI) of 17.9 (7.6–42.2).^14^ Hence, these studies reiterate the importance of monitoring primary care antibiotic prescribing as small changes, such as a rise in clindamycin prescribing, could present serious problems.

This study reports 38% of all cases as non-inpatient and 62% as inpatient. Inpatient CDI incidence decreased over the study period, with slight increases in 2017–18; however, non-inpatient CDI incidence fluctuated throughout this time. A study showing that a reduction of 10% in outpatient antibiotic prescribing could lead to a 17% (95% CI 6%–29.3%) decrease in CA-CDI highlighted a gap in the literature describing population-level impact of antibiotic use on CA-CDI.^31^ Other work, modelling inpatient and outpatient antibiotic stewardship interventions in a regional healthcare network, suggested that a 30% reduction in inpatient and outpatient antibiotic prescribing could lead to a 17% decrease in healthcare-onset (HO) CDI and a 7% reduction in CA-CDI.^32^

### Limitations

A limitation of this study is that the inpatient/non-inpatient definition of CDI cases may not robustly measure the actual exposures to the healthcare system prior to disease presentation; for example, a patient who had recently been hospitalized, discharged and then presented at an emergency department would be classified as a non-inpatient.

### Strengths

Individual-level studies have shown the risks associated with antibiotics and 4C prescribing in the community and hospitals;^14^^,^^30^^,^^33^ however, we believe this to be one of the few to report this association at a population-based ecological level. Although the associations shown at this level of analysis are less striking, the evidence of any relationship between primary care antibiotic prescribing and risk of CDI, particularly after accounting for differing patient demographics, is important.

### Conclusions

In conclusion, this study shows that, even with high variability GP-level prescribing data, an increased risk of CDI can be seen to reflect antibiotic prescribing rates, particularly clindamycin, and demonstrates the continuing importance of antibiotic stewardship by prescribers.

## Funding

The analysis was carried out as part of an Engineering and Physical Sciences Research Council (EPSRC)-funded PhD (F.T.). The data supplied for this studied are routinely collected and accessible upon request. R.R. acknowledges support from the National Institute for Health Research (NIHR) Health Protection Research Unit in Behavioural Science and Evaluation at University of Bristol.

## Transparency declarations

None to declare.

## References

[1] Leffler DA, Lamont JT. *Clostridium difficile* infection. N Engl J Med 2015; 372: 1539–48. 25875259 10.1056/NEJMra1403772

[2] Czepiel J, Dróżdż M, Pituch H et al *Clostridium difficile* infection: review. Eur J Clin Microbiol Infect Dis 2019; 38: 1211–21. 30945014 10.1007/s10096-019-03539-6 PMC6570665

[3] Khanna S, Pardi DS, Aronson SL et al The epidemiology of community-acquired *Clostridium difficile* infection: a population-based study. Am J Gastroenterol 2012; 107: 89–95. 22108454 10.1038/ajg.2011.398 PMC3273904

[4] Public Health Wales Annual CDI Report. https://phw.nhs.wales/services-and-teams/harp/healthcare-associated-infections-hcai/.

[5] PHE. Annual Epidemiological Commentary: Gram-negative bacteraemia, MRSA bacteraemia, MSSA bacteraemia and C. difficile infections, up to and including financial year April 2019 to March 2020. https://assets.publishing.service.gov.uk/government/uploads/system/uploads/attachment_data/file/940716/Annual_epidemiology_commentary_April_2019_March_2020.pdf.

[6] Health Protection Scotland. Healthcare Associated Infection Annual Report 2017. 2018. https://www.hps.scot.nhs.uk/web-resources-container/healthcare-associated-infection-annual-report-2017/.

[7] Banks A, Moore EK, Bishop J et al Trends in mortality following *Clostridium difficile* infection in Scotland, 2010–2016: a retrospective cohort and case–control study. J Hosp Infect 2018; 100: 133–41. 30055220 10.1016/j.jhin.2018.07.023

[8] Robertson C, Pan J, Kavanagh K et al Cost burden of *Clostridioides difficile* infection to the health service: a population based case-control study in Scotland. J Hosp Infect 2020; 106: 554–61. 32717202 10.1016/j.jhin.2020.07.019

[9] Mitchell BG, Gardner A. Mortality and *Clostridium difficile* infection: a review. Antimicrob Resist Infect Control 2012; 1: 20. 22958425 10.1186/2047-2994-1-20 PMC3533881

[10] Cunningham R, Dial S. Is over-use of proton pump inhibitors fuelling the current epidemic of *Clostridium difficile*-associated diarrhoea? J Hosp Infect 2008; 70: 1–6. 10.1016/j.jhin.2008.04.023 18602190

[11] Guh AY, Adkins SH, Li Q et al Risk factors for community-associated *Clostridium difficile* infection in adults: a case-control study. Open Forum Infect Dis 2017; 4: ofx171. 29732377 10.1093/ofid/ofx171 PMC5903408

[12] Ahyow LC, Lambert PC, Jenkins DR et al Bed occupancy rates and hospital-acquired *Clostridium difficile* infection: a cohort study. Infect Control Hosp Epidemiol 2013; 34: 1062–9. 24018923 10.1086/673156

[13] Dingle KE, Didelot X, Quan TP et al Effects of control interventions on *Clostridium difficile* infection in England: an observational study. Lancet Infect Dis 2017; 17: 411–21. 28130063 10.1016/S1473-3099(16)30514-X PMC5368411

[14] Kavanagh K, Pan J, Marwick C et al Cumulative and temporal associations between antimicrobial prescribing and community-associated *Clostridium difficile* infection: population-based case–control study using administrative data. J Antimicrob Chemother 2016; 72: 1193–201. 10.1093/jac/dkw528 27999064

[15] Pan J, Kavanagh K, Marwick C et al Residual effect of community antimicrobial exposure on risk of hospital onset healthcare-associated *Clostridioides difficile* infection: a case–control study using national linked data. J Hosp Infect 2019; 103: 259–67. 31173780 10.1016/j.jhin.2019.05.016

[16] Owens RC Jr, Donskey CJ, Gaynes RP et al Antimicrobial‐associated risk factors for *Clostridium difficile* infection. Clin Infect Dis 2008; 46: S19–31. 18177218 10.1086/521859

[17] NHS Wales. Together for Health. Tackling antimicrobial resistance and improving antibiotic prescribing: a delivery plan for NHS Wales and its partners. 2016. https://gov.wales/sites/default/files/publications/2019-01/together-for-health-tackling-antimicrobial-resistance-and-improving-antibiotic-prescribing.pdf.

[18] Public Health Wales. Antibacterial usage in primary care in Wales. 2018. http://www.wales.nhs.uk/sitesplus/documents/888/Antibacterial%20Usage%20in%20Primary%20Care%20in%20Wales%202013-2017%20%28financial%20years%29.pdf.

[19] All Wales Medicines Strategy Group. Primary Care Antimicrobial Guidelines. 2018. https://awmsg.nhs.wales/files/guidelines-and-pils/primary-care-antimicrobial-guidelines-pdf/.

[20] Health Education and Improvement Wales. Priority areas - stewardship: 4C antimicrobials. https://gpcpd.heiw.wales/clinical/all-wales-national-prescribing-indicators-20-21/priority-areas-stewardship-4c-antimicrobials/.

[21] Public Health Wales. *Clostridium difficile* annual reports. https://phw.nhs.wales/services-and-teams/harp/healthcare-associated-infections-hcai/clostridium-difficile-accordian/clostridium-difficile-annual-reports/.

[22] Statistics for Wales, Statistical First Release. General Medical Services Contract: Quality and Outcomes Framework Statistics for Wales, 2017-18. 2018. https://gov.wales/sites/default/files/statistics-and-research/2019-01/general-medical-services-contract-quality-and-outcomes-framework-financial-year-2017-to-2018.pdf.

[23] Salkind N. Last observation carried forward. In: Encyclopedia of Research Design. SAGE Publications, Inc., 2010.

[24] Public Health England, Public Health Data Science. Technical guide: confidence intervals. 2018. https://fingertips.phe.org.uk/documents/PHDS%20Guidance%20-%20Confidence%20Intervals.pdf.

[25] NICE, BNF. Diabetic foot infections, antibacterial therapy. https://bnf.nice.org.uk/treatment-summary/diabetic-foot-infections-antibacterial-therapy.html.

[26] Macy E, Contreras R. Health care use and serious infection prevalence associated with penicillin “allergy” in hospitalized patients: a cohort study. J Allergy Clin Immunol 2014; 133: 790–6. 24188976 10.1016/j.jaci.2013.09.021

[27] West RM, Smith CJ, Pavitt SH et al ‘Warning: allergic to penicillin’: association between penicillin allergy status in 2.3 million NHS general practice electronic health records, antibiotic prescribing and health outcomes. J Antimicrob Chemother 2019; 74: 2075–82. 31225607 10.1093/jac/dkz127

[28] Blumenthal KG, Lu N, Zhang Y et al Risk of meticillin resistant *Staphylococcus aureus* and *Clostridium difficile* in patients with a documented penicillin allergy: population based matched cohort study. BMJ 2018; 361: k2400. 29950489 10.1136/bmj.k2400 PMC6019853

[29] Thomas C, Boldero R. Antimicrobial stewardship forum: national prescribing indicators. http://www.wales.nhs.uk/sites3/Documents/457/1345%20National%20Prescribing%20Indicators%20Antimicrobial%20Stewardship%20Forum.pdf.

[30] Deshpande A, Pasupuleti V, Thota P et al Community-associated *Clostridium difficile* infection antibiotics: a meta-analysis. J Antimicrob Chemother 2013; 68: 1951–61. 23620467 10.1093/jac/dkt129

[31] Dantes R, Mu Y, Hicks LA et al Association between outpatient antibiotic prescribing practices and community-associated *Clostridium difficile* infection. Open Forum Infect Dis 2015; 2: ofv113. 26509182 10.1093/ofid/ofv113 PMC4551478

[32] Rhea S, Jones K, Endres-Dighe S et al Modeling inpatient and outpatient antibiotic stewardship interventions to reduce the burden of *Clostridioides difficile* infection in a regional healthcare network. PLoS One 2020; 15: e0234031. 32525887 10.1371/journal.pone.0234031 PMC7289388

[33] Brown KA, Khanafer N, Daneman N et al Meta-analysis of antibiotics and the risk of community-associated *Clostridium difficile* infection. Antimicrob Agents Chemother 2013; 57: 2326–32. 23478961 10.1128/AAC.02176-12 PMC3632900

